# Early maladaptive schemas as predictors of depressive symptoms and treatment success in an outpatient rehabilitation sample

**DOI:** 10.3389/fpsyt.2026.1698633

**Published:** 2026-01-28

**Authors:** Alexandra Schosser, Daniela Fischer-Hansal, Miriam Traugott-Suchomel, Birgit Senft

**Affiliations:** 1 Faculty of Medicine, Sigmund Freud Private University Vienna, Vienna, Austria; 2 Center for Mental Health Leopoldau, BBRZ MED, Vienna, Austria

**Keywords:** cognitive behavioral therapy, depression, early maladaptive schema (EMS), psychotherapy, rehabilitation, risk factors, schema therapy, treatment outcome

## Abstract

**Background and objectives:**

Early maladaptive schemas (EMSs) are cognitive–emotional patterns linked to depression. Their role in psychiatric rehabilitation and treatment success remains unclear. This study examines the association between EMSs and depressive symptom severity at admission and its impact on treatment outcomes.

**Materials and methods:**

A total of 2,568 patients in a 6-week outpatient psychiatric rehabilitation program in Austria were assessed. EMSs were measured using the Young Schema Questionnaire-Short Form 3 (YSQ-S3), and depressive symptoms were measured using the Beck Depression Inventory-Second Edition (BDI-2). Multiple regression analyses determined the predictive value of EMSs on symptom severity and treatment response. Cluster analyses identified schema profiles and their relation to treatment success.

**Results:**

Higher EMS scores, especially in *Disconnection/Rejection* and *Impaired Autonomy/Performance*, correlated with higher depression levels at admission. Regression models explained 46% of symptom variance. However, EMSs had limited predictive value for treatment success, with small effects for *Dependence/Incompetence* and *Subjugation*. Cluster analyses showed that patients with high EMSs had more severe symptoms but similar clinical improvement.

**Discussion:**

EMSs showed a significant influence on the severity of depressive symptoms at admission. However, their impact on treatment success was limited; namely, similar clinical improvement was found in patients with high EMSs.

## Background

### Early maladaptive schemas in psychiatric disorders

Early maladaptive schemas (EMSs) reflect stable, trait-like beliefs that underlie personality (1–3). They are thought to develop through an interaction of early negative childhood experiences (e.g., rejection, aggressive parents, and lack of emotional security) and a person’s temperament. EMSs thus are deeply ingrained cognitive–emotional patterns that develop in response to unmet core emotional needs during childhood and persist into adulthood (3). These schemas influence how individuals perceive themselves, others, and the world, often leading to negative self-beliefs and dysfunctional coping strategies (4). EMSs are considered cognitive risk factors for various psychological disorders, particularly depression (5), and are core concepts of schema therapy (3). Although EMSs were primarily developed for their assessment in personality disorders, they were shown to exhibit marked relationships to clinical disorders—e.g., anorexia nervosa and unrelenting standards, major depressive disorder and social isolation, or attention deficit hyperactivity disorder and insufficient self-control (6). Nevertheless, a recent systematic review and meta-analysis including 27 studies mainly investigating depression (N = 8), borderline personality disorder (N = 5), and obsessive-compulsive disorder (N = 5) pointed out that mental disorders are not characterized by specific EMSs (7). However, heterogeneity between studies was high, complicating the interpretation of these findings.

### Psychiatric rehabilitation in Austria

Psychiatric rehabilitation promotes recovery, full integration into the community, and an improved quality of life for people diagnosed with a mental illness that seriously impairs their ability to live meaningful lives. In Austria, the majority of patients treated in psychiatric rehabilitation clinics suffer from affective and/or anxiety disorders, with a high proportion of the conditions showing a chronic course of disease (8).

The current study focuses on EMSs as predictors of depressive symptoms and treatment success in the context of a 6-week outpatient psychiatric rehabilitation program [the so-called World Health Organization (WHO) “Phase 2 Rehabilitation”] (9). Psychiatric rehabilitation in Austria was initially established as an inpatient treatment program in 2002, followed by outpatient treatment programs from 2010.

In general, psychiatric rehabilitation in Austria has to be carried out in accordance with the guidelines of the Social Pension Insurance Institution (PVA), bearing the scientific advantage of a very standardized treatment program. By the time the current study was performed, according to the PVA, 142 treatment units of 50 minutes each were provided within 6 weeks of rehabilitation, consisting of 54 units of psychotherapy, 36 units of occupational therapy, 18 units of physiotherapy, 12 units of social work, 15 units of flexible choice of therapy, and seven units of other non-therapeutic interventions including medical examinations. Of note, the rehabilitation program did not include schema therapy.

### Early maladaptive schemas and depression

Meta-analytic evidence suggests that EMSs are strongly associated with depression in adulthood. A systematic review and meta-analysis (10) based on 51 studies found that all 18 EMSs showed positive correlations with depression symptoms.

Cognitive theories of depression suggest that EMSs contribute to the onset and maintenance of depressive episodes by reinforcing negative self-perceptions and maladaptive coping mechanisms (11). These schemas often lead to excessive self-criticism, social withdrawal, and difficulties seeking support, which in turn perpetuates depression symptomatology (12). Schema therapy, which targets EMSs through cognitive restructuring and experiential techniques, has shown promise in reducing depression severity, particularly in individuals with chronic or treatment-resistant depression (13).

Given the strong link between EMSs and depression, addressing these underlying cognitive vulnerabilities in therapy may enhance treatment outcomes and prevent relapse. Future research should focus on longitudinal studies to better understand the causal relationship between EMSs and depressive disorders.

A meta-analysis (24 studies, *N* = 13,635 participants, ø age = 19.49) of adolescents and young adults showed a significant correlation between overall EMSs and depression to the extent of a large effect size (r = 0.56). Higher levels of EMSs were associated with higher levels of depression. Moderator analyses showed no impact of age and gender on the correlations. At the domain level, there were particularly strong associations between depression and D1 Disconnection/Rejection (r = 0.49) and D2 Impaired Autonomy/Performance (r = 0.47), while the associations with D3 Impaired Limits (r = 0.36), D4 Other-Directedness (r = 0.40), and D5 Hypervigilance (r = 0.31) were somewhat lower (14).

In a meta-analysis (51 studies, *N* = 17,830, mean age from 18.50 to 69.22, often clinical samples) performed in adults, significant correlations were found between all EMSs and depression, from small to large effect sizes (10). Strong correlations were found between depression and DS Defectiveness/Shame (r = 0.50) and SI Social Isolation (r = 0.53). Moderate correlations were found between depression and ED Emotional Deprivation (r = 0.42), AB Abandonment (r = 0.46), MA Mistrust/Abuse (r = 0.44), FA Failure (r = 0.46), DI Dependence/Incompetence (r = 0.46), VH Vulnerability to Harm (r = 0.47), EM Enmeshment (r = 0.33), IS Insufficient Self-Control (r = 0.43), SB Subjugation (r = 0.46), AS Approval-Seeking (r = 0.30), EI Emotional Inhibition (r = 0.40), NP Negativity (r = 0.49), and PU Punitiveness (r = 0.36). Small correlations were found between depression and ET Entitlement (r = 0.23), SS Self-Sacrificing (r = 0.27), and US Unrelenting Standards (r = 0.25). The authors concluded that, in particular, those schemas played a significant role in the development of depression symptoms, which give rise to a feeling of being unloved/unlovable or inadequate. Again, heterogeneity across studies may complicate the interpretation of these findings and may explain inconsistent findings (10).

The predictive value of the schemas for treatment response was investigated in several studies. In a naturalistic study performed in a heterogeneous outpatient sample, domain Other Directness proved to be a significant predictor of greater symptom reduction (15). Further, two schemas, Failure (FA) and Emotional Inhibition (EI), also proved to be negative predictors for treatment success in obsessive-compulsive disorder (16). A study examining the effect of an integrative psychodynamic inpatient therapy without explicit focus on EMSs in a major depression sample (17) revealed that EMSs were significantly reduced in three out of five schema domains. Importantly, the reduction of symptom distress during treatment was strongly associated with a reduction in EMSs of the schema domain “Impaired Autonomy/Performance”. The authors conclude that changes in EMSs are highly relevant for changes in symptom distress, with EMSs being changed not only by schema therapy but also by other therapy approaches.

Finally, Sunde et al. (18) were able to show in patients with obsessive-compulsive disorder that high pre-treatment EMS scores were associated with a higher level of symptoms at the follow-up point 1 year after the end of treatment and that non-recovered patients had significantly higher EMS scores at the start of treatment. Since pre-treatment EMSs were clearly associated with a poorer long-term outcome, the authors recommend a combined treatment that also addresses EMSs in cases of higher EMSs.

### Aim of the study

The aim of the current study was to analyze the influence of EMSs (schema scores) on symptom burden at admission to an outpatient rehabilitation program, as well as on the change in symptom burden until the end of rehabilitation. In addition, cluster analyses were applied to gain insight into empirically identified subgroups based on EMSs and how these subgroups differentiate with respect to (depressive) symptomatology and treatment success. The study was designed as a prospective exploratory study.

## Material and methods

### Treatment conditions

Treatment was performed as a standardized 6-week multi-professional rehabilitation program, composed of group and individual psychotherapy, occupational therapy, physiotherapy, social work, and weekly psychiatrist’s consultations. All treatments were based on Cognitive Behavioral Therapy (CBT), with the majority of psychotherapists having expertise in schema therapy and thus using schema therapeutic treatment elements in individual and group psychotherapy sessions. All individuals were asked to complete questionnaires upon admission and at the end of the 6-week rehabilitation program. At admission, among others, their medical records and occupational status within the 12 months before admission were also collected.

### Sample description

Of all rehabilitation patients treated at the “Center for Mental Health BBRZ-Med Vienna-LEOpoldau” between 2014 and 2023, Young Schema Questionnaire-Short Form 3 (YSQ-S3) questionnaires were available for a subgroup of 2,601 patients at admission to the rehabilitation program. Cases involving discontinued rehabilitation or language problems were not included in the current study. These two groups accounted for 32% of the total sample. Cases with too many missing values in the YSQ-S3 were excluded from the analyses (admission, N = 17; end of rehabilitation, N = 16). The final sample consisted of 2,568 patients (34.1% male and 65.9% female), and the average age was 48.15 years [standard deviation (SD) = 10.16]. The majority of patients (89.1%) treated in this ambulant psychiatric rehabilitation setting suffered from affective (63.4%) or anxiety disorders (25.7%) as main diagnoses, and a further 6.6% had a personality disorder as main diagnosis. A high proportion of rehabilitands were diagnosed with one (54.1%) or more (19.5%) psychiatric comorbidities. As for the occupational status, only 29.9% of rehabilitation patients were employed, 52.5% were unemployed, and 15.6% received rehabilitation allowance. The detailed sample description is shown in **Table 1**.

**Table 1:** Sample description.

Total	*N* = 2,568	100%
Age	M = 48.15	SD = 10.16
Male	876	34.1%
Female	1,692	65.9%
Education	*N*	%
Secondary school maximum	487	19.0
Apprenticeship training	804	31.3
Further school education	709	27.6
College/university	319	12.4
Others	235	9.2
No information	14	0.5
Marital status	*N*	%
Never married	844	32.9
Currently married	784	30.5
Divorced	540	21
Widowed	28	1.1
Cohabitating	364	14.2
No information	8	0.3
Own children	1,452	56.5
Work status	*N*	%
Employed	768	29.9
Unemployed	1,348	52.5
Rehabilitation allowance/retired	401	15.6
Others	19	0.7
No information	32	1.3
Diagnoses (ICD-10)	*N*	%
F3 Mood (affective) disorders	1,628	63.4
F4 Neurotic-, stress-related and somatof. disorders	661	25.7
F6 Disorders of adult personality and behavior	169	6.6
Others	110	4.3
At least one psychiatric comorbidity	1,389	54.1
At least two psychiatric comorbidities	501	19.5

M, mean; SD, standard deviation; ICD, International Statistical Classification of Diseases and Related Health Problems, 10th Revision. Other diagnoses: F00–F09 Organic, including symptomatic, mental disorders, *N* = 3; F10–F19 Mental and behavioral disorders due to psychoactive substance use, *N* = 8; F20–F29 Schizophrenia, schizotypal and delusional disorders, *N* = 44; F50–F59 Behavioral syndromes associated with physiological disturbances and physical factors, *N* = 1; F70–F79 Mental retardation, *N* = 2; F80–F89 Disorders of psychological development, *N* = 3; F90–F98 Behavioral and emotional disorders with onset usually occurring in childhood and adolescence, *N* = 21; Z73 Problems related to life-management difficulty, *N* = 4; Z76 Persons encountering health services in other circumstances *N* = 24.

### Measurements

#### Young Schema Questionnaire-Short Form 3

The YSQ-S3 is a self-assessment including 90 items. The 18 early maladaptive schemas were assessed with five items each and can be assigned to one of the five schema domains each (19). The schema domains consist of the following EMSs: D1: Disconnection: AB: Abandonment/Instability, MA: Mistrust/Abuse, ED: Emotional Deprivation, DS: Defectiveness/Shame, and SI: Social Isolation/Alienation; D2: Impaired Autonomy and Achievement: DI: Dependence/Incompetence, VH: Vulnerability to Harm and Illness, EM: Enmeshment/Undeveloped Self, and FA: Failure; D3: Impaired Limits: ET: Entitlement/Grandiosity and IS: Insufficient Self-Control/Self-Discipline; D4: Other-Directness: SB: Subjugation, SS: Self-Sacrifice and AS: Approval-Seeking/Recognition-Seeking; and D5: Exaggerated Vigilance and Inhibition NP: Negativity: EI: Emotional Inhibition, US: Unrelenting Standards/Hypercriticalness, and PU: Punitiveness. Higher schema scores indicate stronger endorsement of a given schema. The German version of the Young Schema Questionnaire was published in 2007 (20), and validation was performed (21). The YSQ is available in multiple languages and includes sufficient test statistical values; a validation sample for the Austrian population has not yet been implemented.

#### Beck Depression Inventory

The Beck Depression Inventory (BDI-2) (22) is a measurement of depression severity that was derived from clinical observations about the attitudes and symptoms displayed frequently by depressed psychiatric patients and infrequently by non-depressed psychiatric patients. The clinical observations were consolidated systematically into 21 symptoms and attitudes (mood, pessimism, sense of failure, lack of satisfaction, guilt feelings, sense of punishment, self-dislike, self-accusation, suicidal wishes, crying, irritability, social withdrawal, indecisiveness, distortion of body image, work inhibition, sleep disturbance, fatigability, loss of appetite, weight loss, somatic preoccupation, and loss of libido), which could be rated from 0 to 3 in terms of intensity. Originally, the Beck Depression Inventory-Second Edition (BDI-2) was designed to be administered by trained interviewers; however, it is most often self-administered. The cut-off scores are 0 to 10 for none or minimal depression, 11 to 17 for mild to moderate depression, and 18 to 63 for severe depression. The BDI-2 was developed in accordance with the Diagnostic and Statistical Manual of Mental Disorders, Fourth Edition (DSM-IV) and features good test statistical values. Standard values are available for depressed and healthy individuals; significant changes are defined as a cut-off of 8 points (22). The internal consistency within the current study was α = 0.902, and the BDI-2 was defined as the primary outcome.

#### Further data ascertainment

Relevant sociodemographic data were collected at the time of admission to rehabilitation by means of self-provided items. The diagnoses were determined in accordance with the International Statistical Classification of Diseases and Related Health Problems, 10th Revision (ICD-10) upon discharge. The overall success of rehabilitation was assessed by the rehabilitation patients using a 4-point scale (very successful, partially successful, hardly successful, and not successful). The achievement of individual goals was also assessed from the perspective of the rehabilitation patients on a 4-point scale (more than achieved, achieved, partially achieved, and not achieved).

Treatment response was thus assessed on the basis of changes in the BDI-2, the global self-assessment of treatment success, and the evaluation of the achievement of the first rehabilitation goal.

### Statistical analyses

Statistical analyses were performed using IBM SPSS Statistics Version 29 and Tableau. In the course of descriptive statistics, means (M) and SDs, as well as frequencies and %, were reported.

Multiple linear regressions (backward method) were calculated to evaluate the influence of the 18 EMSs and five domains (predictors, independent variables) on depression symptoms (BDI-2, dependent variable, criterion). These predictors were also used to predict the criterion of numerical differences in depression symptoms (BDI-2) between the beginning (admission) and end of rehabilitation. The backward method was chosen in order to gradually exclude non-significant predictors and obtain a final model with relevant predictors and a corresponding variance explanation. The presence of multicollinearity was tested using the variance inflation factor (VIF), tolerance, and condition index; all models showed sufficient test statistics.

To characterize rehabilitation patients with regard to schemas, a cluster analysis according to Ward, with the Euclidean distance, was performed. Using this method, cases can be broken down into subgroups that are homogeneous within themselves and heterogeneous in relation to each other, thereby enabling exploratory structures in the data to be uncovered. In order to evaluate the changes in the BDI-2 scores depending on cluster membership, an analysis of variance with repeated measures was conducted. Cohen’s *d* was used as an effect size to measure the change (*d* = 0.20 = small, *d* = 0.50 = medium, and *d* = 0.80 = large) (23). Relationships between categorical data (cluster-membership/goal attainment/treatment success) were examined by applying a Chi^2^ statistic; the effect size for ω was calculated using the formula $\omega =\sqrt{\frac{Chi^{2}}{N}}$ and interpreted as follows: ω = 0.10 = small, ω = 0.30 = medium, and ω = 0.50 = large (24). A value of *p* < 0.05 was considered to be statistically significant.

## Results

The depression symptom burden, as assessed using the BDI-2, was high at admission to the rehabilitation program and had an average BDI-2 score of 26.48 (SD = 11.17). The raw scores in the BDI-2 are interpreted as follows: 0–8, none; 9–13, minimal; 14–19, mild; 20–28, moderate; and 29–63, severe depression.

### Predictive value of EMSs for depression symptoms at admission

Multiple linear regression (backward method) indicated a significant model for predicting depression symptoms based on the schema scores (*R* = 0.68, *R*^2adj^ = 0.46, *F*_(11)_ = 198.06, *p* < 0.001). Eleven out of the 18 schemas assessed using the YSQ-S3 explained a high proportion (46%) of the variance in depression symptoms at admission. With higher levels in the schemas Entitlement/Grandiosity (ET) and Approval-Seeking/Recognition-Seeking (AS), lower levels of depression were associated. For the remaining nine schemas, higher levels were associated with higher levels of depressiveness (**Table 2**). Two EMSs, Dependence/Incompetence (DI) and Vulnerability to Harm and Illness (VH), proved to be the strongest predictors. No significant predictive value was found for EMSs Enmeshment/Undeveloped Self (EM; *B* = −0.011, *p* = 0.536), Failure (FA; *B* = −0.011, *p* = 0.640), Abandonment/Instability (AB; *B* = −0.014, *p* = 0.484), Punitiveness (PU; *B* = 0.017, *p* = 0.406), Mistrust/Abuse (MA; *B* = 0.022, *p* = 0.334), Subjugation (SB; *B* = −0.023, *p* = 0.313), and Social Isolation/Alienation (SI; *B* = 0.030, *p* = 0.181).

**Table 2:** Predictors for depression symptoms at start of rehabilitation.

Model 8	Unstandard. coefficients		Standard. coefficients	t	Sig.	95.0% confidence interval for B	
Dependent variable: BDI-2 at admission	B	Std. error	Beta			Lower bound	Upper bound
(Constant)	2.19	0.76		2.89	0.004	0.70	3.67
ED: Emotional Deprivation	0.58	0.16	0.07	3.73	<0.001	0.28	0.89
DS: Defectiveness/Shame	0.97	0.20	0.11	4.77	<0.001	0.57	1.36
DI: Dependence/Incompetence	2.37	0.22	0.24	10.80	<0.001	1.94	2.80
VH: Vulnerability to Harm and Illness	1.32	0.21	0.15	6.37	<0.001	0.92	1.73
ET: Entitlement/Grandiosity	-0.81	0.23	-0.06	-3.48	0.001	-1.26	-0.35
IS: Insufficient Self-Control/Self-Discipline	1.18	0.22	0.11	5.44	<0.001	0.76	1.61
SS: Self-Sacrifice	0.99	0.16	0.10	6.26	<0.001	0.68	1.30
AS: Approval-Seeking/Recognition-Seeking	-0.96	0.19	-0.10	-5.15	<0.001	-1.33	-0.59
NP: Negativity	0.94	0.21	0.11	4.45	<0.001	0.53	1.36
EI: Emotional Inhibition	0.46	0.19	0.05	2.45	0.014	0.09	0.82
US: Unrelenting Standards/Hypercriticalness	0.66	0.19	0.07	3.45	0.001	0.29	1.04

BDI-2, Beck Depression Inventory-Second Edition.

It can be concluded that clear associations between the EMSs and the depression symptoms were found. Patients with higher schema scores (EMSs) were more depressed at the start of rehabilitation (admission); the explained variance was notable at 46%.

The second multiple linear regression (backward method) indicated a significant model for predicting depression symptoms based on the schema domains (*R* = 0.64, *R*^2adj.^ = 0.41, *F*_(3)_ = 595.21, *p* < 0.001). The five schema domains explained a high proportion (41%) of the variance in depression symptoms at admission. Two domains, D3 Impaired Limits (*B* = −0.009, *p* = 0.653) and D4 Other Directness (*B* = −0.031, *p* = 0.177), proved to be insufficiently predictive for depression symptoms. D1 Disconnection (*B* = 1.561, *p* < 0.001), D2 Impaired Autonomy and Achievement (*B* = 4.149, *p* < 0.001), and D5 Exaggerated Vigilance and Inhibition (*B* = 2.510, *p* < 0.001) remained as significant predictors in the model. Higher domain scores were associated with higher levels of depression.

### Predictive value of EMSs and domains on rehabilitation success

For the prediction of the reduction of depression symptoms (difference in BDI-2 raw scores from admission to discharge), a significant regression model was found, but with a small proportion of variance explained (*R* = 0.15, *R*^2adj^ = 0.02, *F*_(6)_ = 9.54, *p* < 0.001). Of the EMSs that remained in the backward model, only Dependence/Incompetence (DI), Subjugation (SB), Approval-Seeking/Recognition-Seeking (AS), and Punitiveness (PU) significantly predicted the difference in BDI-2 (*p* ≤ 0.05). Higher values in the SB, AS, and PU schemes were accompanied by higher values in the reduction of depression symptoms. Higher values in DI were associated with a lower reduction in depression symptoms. Two EMSs, Entitlement/Grandiosity (ET) and Insufficient Self-Control/Self-Discipline (IS), remained in the model, but the influence was not sufficiently certain (**Table 3**).

**Table 3:** Predictors for the prediction of symptom reduction in BDI-2 between admission and discharge.

Model 13	Unstandard. coefficients		Standard. coefficients	T	Sig.	95.0% confidence interval for B	
Dependent variable: difference in BDI-2 scores (discharge–admission)	B	Std. error	Beta			Lower bound	Upper bound
(Constant)	4.69	0.74		6.34	<0.001	3.24	6.15
DI: Dependence/Incompetence	−0.48	0.23	−0.06	−2.08	0.037	−0.93	−0.03
ET: Entitlement/Grandiosity	−0.45	0.25	−0.04	−1.83	0.067	−0.93	0.03
IS: Insufficient Self-Control/Self-Discipline	0.42	0.24	0.05	1.75	0.008	−0.05	0.89
SB: Subjugation	0.73	0.21	0.10	3.55	<0.001	0.33	1.13
AS: Approval-Seeking/Recognition-Seeking	0.41	0.20	0.05	2.05	0.004	0.02	0.79
PU: Punitiveness	0.51	0.21	0.06	2.36	0.018	0.09	0.93

BDI-2, Beck Depression Inventory.

Most of the EMSs had no predictive value at all for the reduction of depression symptoms. This held true for EMSs Failure (FA), Emotional Deprivation (ED), Defectiveness/Shame (DS), Mistrust/Abuse (MA), Abandonment/Instability (AB), Self-Sacrifice (SS), Enmeshment/Undeveloped Self (EM), Emotional Inhibition (EI), Unrelenting (US) Standards/Hypercriticalness, Social Isolation/Alienation (SI), Negativity (NP), and Vulnerability to Harm and Illness (VH). Detailed statistical analyses can be found in **Supplementary Table S1**.

There was no linear and meaningful association between EMS scores and the reduction of depression symptoms. The majority of EMSs had no predictive value for changes in the BDI-2. The variance explanation was too low to assume an impact of the schema scores on the change.

A second regression analysis to predict the change in the BDI-2 was carried out at the domain level and showed a significant regression model, but also with a small proportion of variance explained (*R* = 0.13, *R*^2adj^ = 0.02, *F*_(1)_ = 43.88, *p* < 0.001). By applying the backward method, only D4 Other-Directness (*B* = −1.366, *p* < 0.001) remained in the model as a significant predictor. Domains D1 Disconnection (*B* = 0.004, *p* = 0.871), D2 Impaired Autonomy and Achievement (*B* = −0.001, *p* = 0.966), D3 Impaired Limits (*B* = −0.001, *p* = 0.977), and D5 Exaggerated Vigilance and Inhibition (*B* = 0.040, *p* = 0.147) had no significant predictive value for the difference in BDI-2.

### Results of the cluster analysis of the schema values

**Figure 1:**
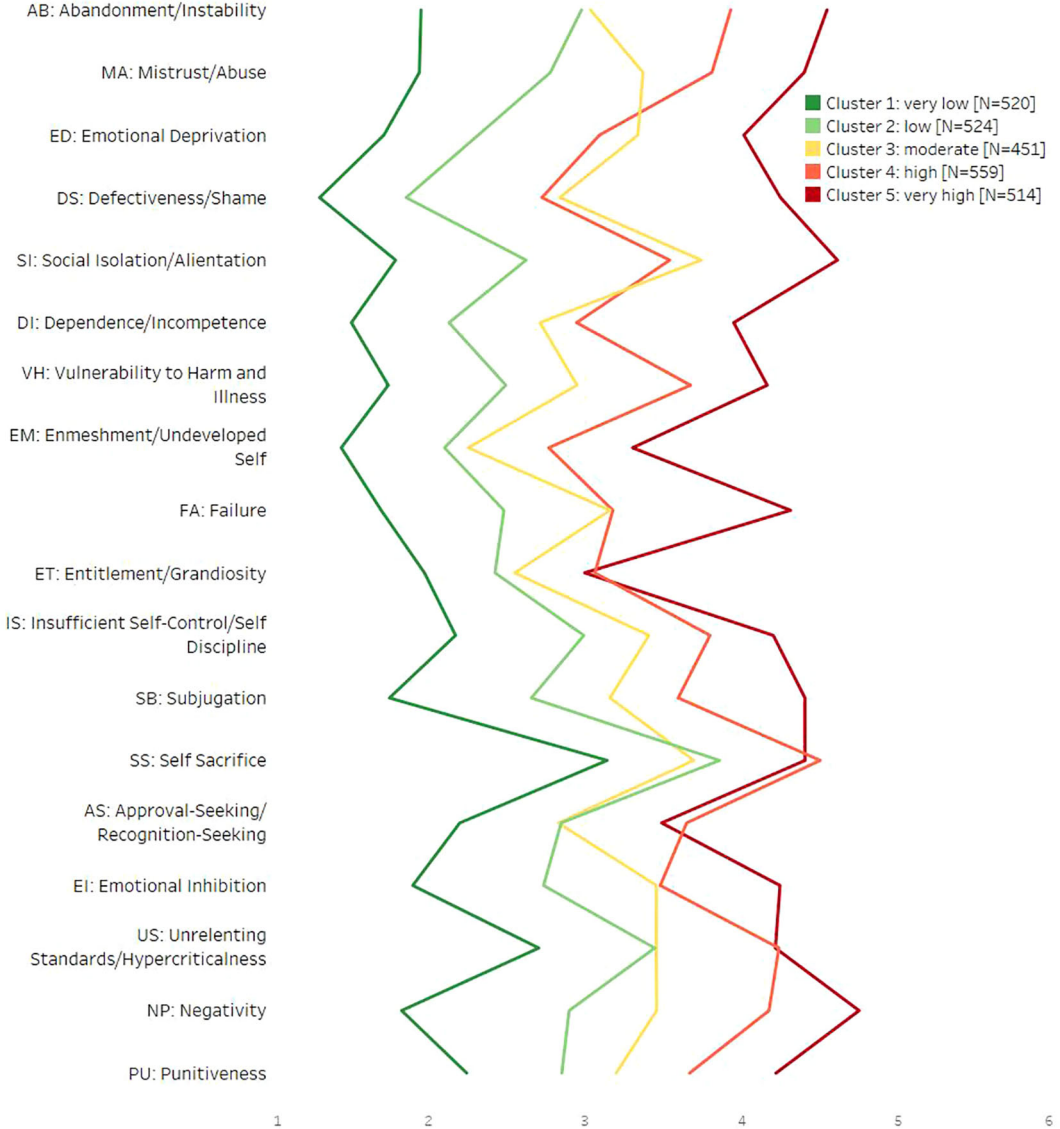
Average EMS values depending on cluster membership. EMS, early maladaptive schema.

Of the possible results of cluster analysis, the five-cluster solution was selected since these were clearly arranged, and the clusters contained an acceptable number of patients each. **Figure 1** shows the peculiarities of the cluster.

Cluster 1 “very low” (20.2%) showed low mean values in all schemas, and only for the schema self-sacrifice (SS) was the mean value above 3.0.

Cluster 2 “low” (20.4%) showed a similar profile; however, for half of the schemas, the mean values (*M*) were one score above the first cluster. The highest mean values were found for the schemes Self-Sacrifice (SS; *M* = 3.86) and Unrelenting Standards (US; *M* = 3.44).

Cluster 3 “moderate” (17.6%) was characterized by a high level of Social Isolation (SI; *M* = 3.74), Emotional Deprivation (ED; *M* = 3.33), Emotional Inhibition (EI; *M* = 3.45), and Negativity (NP; *M* = 3.45) and by lower values in schemes Abandonment (AB; *M* = 3.03), Enmeshment (EM; *M* = 2.25), Entitlement (ET; *M* = 2.55), Approval-Seeking (AS; *M* = 2.83), and Unrelenting Standards (US; *M* = 3.45).

Cluster 4 “high” (21.8%) showed the second-highest mean values for most schemas. In particular, high mean values were found for schemes Self-Sacrifice (SS; *M* = 4.50), Negativity (NP; *M* = 4.17), and Approval-Seeking (AS; *M* = 3.65). In relation to the position of this cluster, schemes Emotional Deprivation (ED; *M* = 3.09), Defectiveness/Shame (DS; *M* = 2.72), and Emotional Inhibition (EI; *M* = 3.48) were low (equal to or lower than in Cluster 3).

Cluster 5 “very high” (20.0%) showed the highest mean values for nearly all schemes, partly with a significant interval. The highest mean values were found for schemes Negativity (NP; *M* = 4.75), Social Isolation (SI; *M* = 4.61), and Abandonment (AB; *M* = 4.54).

### Rehabilitation success for patients with differing schema profiles (cluster)

When analyzing the rehabilitation success, defined as a reduction in symptom burden, it was found that the schema scores could not predict changes in BDI-2 scores. However, the analysis of variances with repeated measures for the interaction time * cluster showed a significant result (*F*_[4, 2563]_ = 10.11, *p* < 0.001). However, the effect of the interaction was small, with *f* = 0.127.

**Figure 2:**
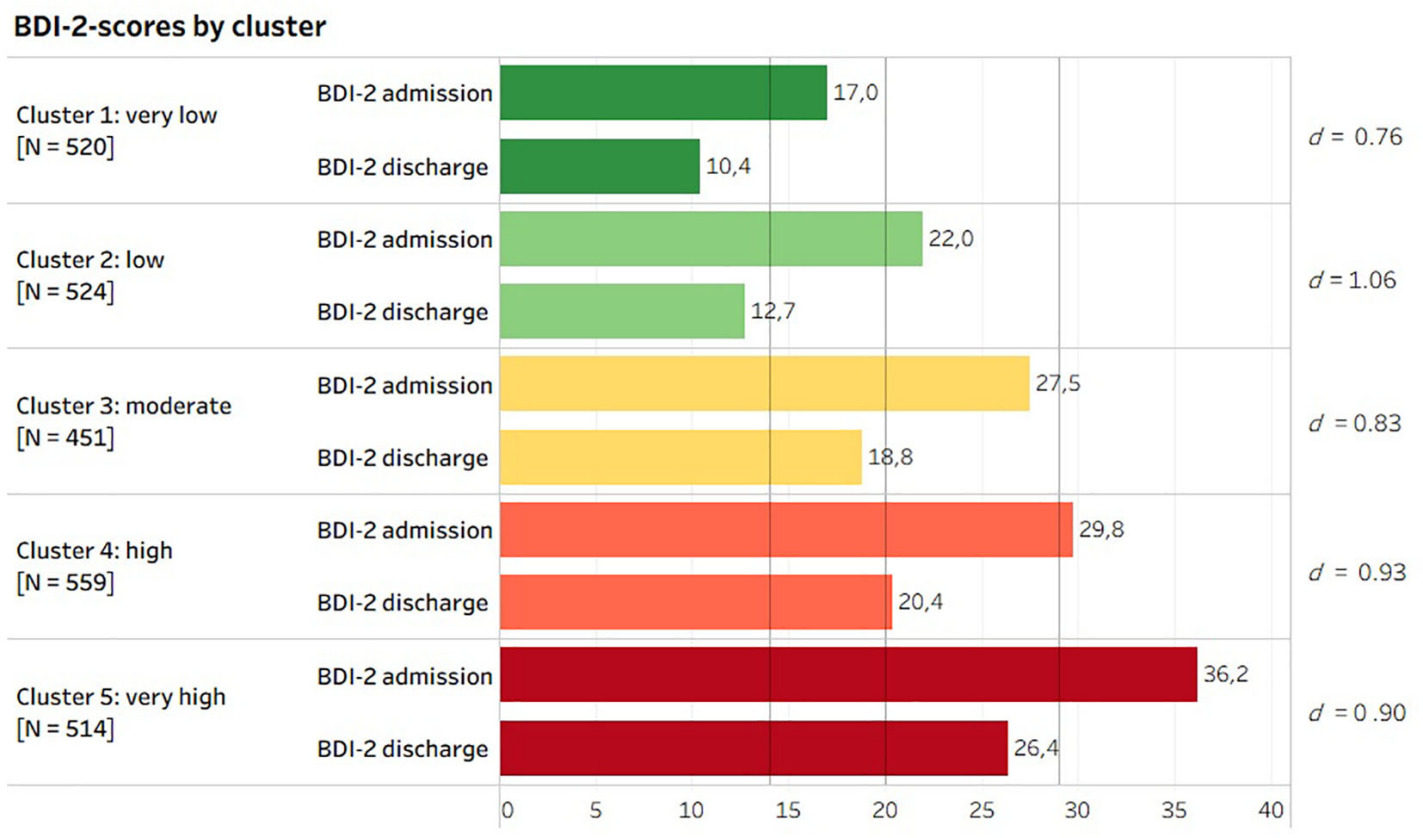
BDI-2 values at admission and discharge depending on cluster affiliation with Cohen’s *d* effect size. Legend: BDI-2 scores: <14 = minimal, 14–19 = mild, 20–28 = moderate, and 29–63 = severe depression symptoms. BDI-2, Beck Depression Inventory.

It was shown that patients assigned to differing clusters start with very diverging base levels (BDI-2), thus finishing rehabilitation with very different depression symptoms (**Figure 2**). Cluster 1 showed lower depression scores as measured by BDI-2 at admission than clusters 3, 4, and 5 at discharge. Cluster 5 with the highest schema scores still showed a BDI-2 mean score of 26.39 (*SD* = 12.27) at discharge, corresponding to severe depression. Clusters 3 and 4 showed BDI-2 scores at admission within the higher medium range; however, they decreased at the time of discharge to the medium to lower range of depression scores as measured by BDI-2. The two clusters with low schema scores, clusters 1 and 2, corresponded to mild to moderate depression at admission, and a decrease to minimal depression symptoms at discharge. However, in all clusters, large effect sizes were evident (**Figure 2**).

**Figure 3:**
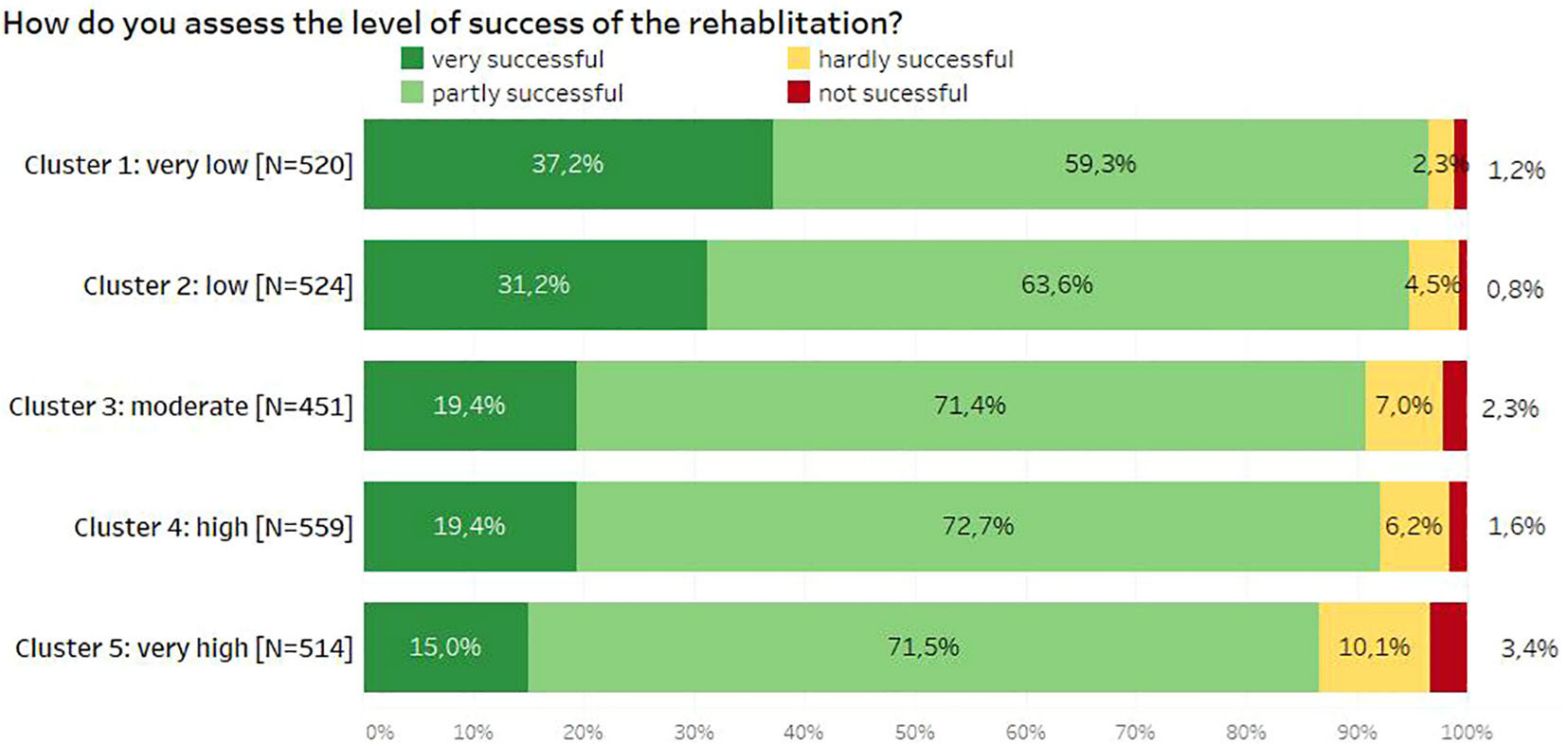
Self-assessment of rehabilitation success depending on cluster membership.

**Figure 4:**
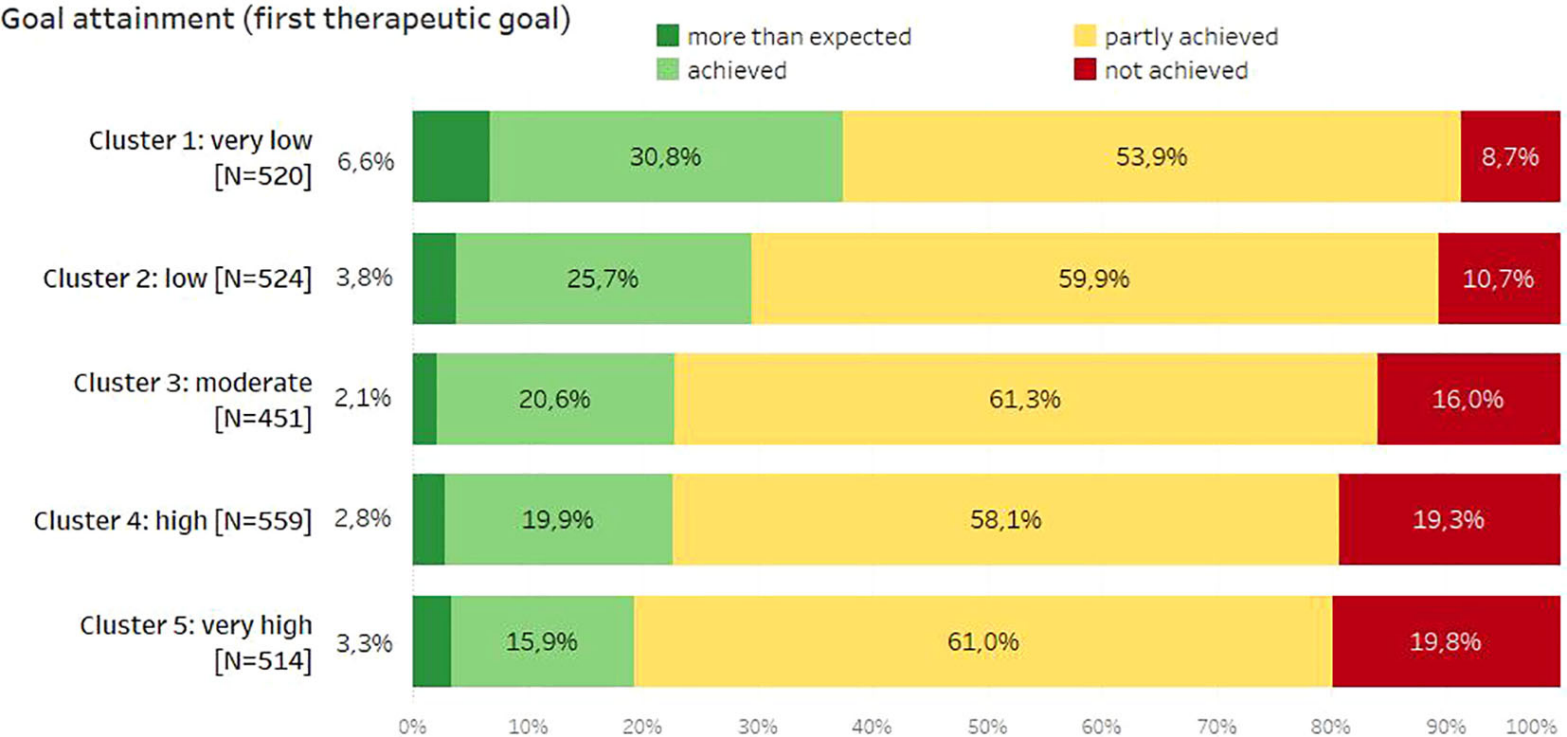
Self-assessment of goal attainment depending on cluster membership.

A significant association between self-assessment of rehabilitation success and cluster affiliation was found (Xi^2[12]^ = 123.38, *p* < 0.001), with a small-to-medium effect size ω of 0.22 (**Figure 3**). The same held true for the attainment of therapy goals by the patients (calculated for the first therapeutic goal assessed by patients upon discharge), being significantly associated with cluster affiliation as well (Xi^2[12]^ = 82.35, *p* < 0.001), again with a small effect size ω of 0.18 (**Figure 4**).

This means that patients with higher schema scores were significantly more critical in their assessment of the success of rehabilitation and the achievement of therapy goals. Patients with higher schema scores rated the success of rehabilitation and the achievement of therapy goals significantly lower. Compared to cluster 5, more than twice as many patients assigned to clusters 1 and 2 rated the success of rehabilitation as very high. In cluster 5, however, the proportion of rehabilitation patients who reported little or no success in rehabilitation was the highest. In terms of therapy goals, almost twice as many goals were achieved in cluster 1 as in cluster 5.

Finally, the clusters were examined for significant differences in patient characteristics. Significant differences were found for all variables, but all effects were small (**Table 4**). In terms of trends, rehabilitation patients in cluster 5 tended to be slightly younger, and the proportion of women was slightly higher in clusters 2, 4, and 5. Clusters 3 and 5 showed a slightly lower proportion of academics, and clusters 3, 4, and 5 were characterized by higher proportions of patients with personality disorders and higher proportions of comorbidities.

**Table 4:** Patient characteristics depending on cluster membership.

Patient characteristics	Cluster 1 EMS very low		Cluster 2 EMS low		Cluster 3 EMS moderate		Cluster 4 EMS high		Cluster 5 EMS very high	
	*N* = 520		*N* = 524		*N* = 451		*N* = 559		*N* = 514	
Age (*p* < .001; *η*^2^ = 0.02)	49.92	9.57	48.61	10.21	48.51	9.56	47.98	10.19	45.75	10.75
Male (*p* = .006; ω = 0.08)	202	38.80%	167	31.90%	173	38.40%	179	32.00%	155	30.20%
Female	318	61.20%	357	68.10%	278	61.60%	380	68.00%	359	69.80%
Education (*p* = .007; ω = 0.15)	*N*	%	*N*	%	*N*	%	*N*	%	*N*	%
											Secondary school maximum	91	17.40%	97	18.50%	80	17.80%	103	18.50%	116	22.50%
Apprenticeship training	166	32.00%	138	26.30%	171	37.90%	167	29.90%	162	31.50%
Further school education	142	27.30%	146	27.90%	114	25.30%	157	28.10%	150	29.20%
College/university	74	14.20%	80	15.30%	43	9.50%	79	14.10%	43	8.40%
Others	44	8.50%	62	11.80%	42	9.30%	48	8.50%	39	7.60%
No information	3	0.60%	1	0.20%	1	0.20%	5	0.90%	4	0.80%
Marital status (*p* < .001; ω = 0.13)	*N*	%	*N*	%	*N*	%	*N*	%	*N*	%
											Never married	157	30.20%	155	29.60%	166	36.80%	168	30.10%	198	38.50%
Currently married	185	35.60%	178	34.00%	122	27.10%	174	31.10%	125	24.30%
Divorced	92	17.70%	104	19.80%	101	22.40%	121	21.70%	122	23.70%
Widowed	8	1.50%	3	0,6%	6	1.30%	10	1.80%	1	0.20%
Cohabitating	78	15.00%	84	16.00%	54	12.00%	83	14.80%	65	12.70%
No information					2	0.40%	3	0.50%	3	0.60%
Own children (*p* = .020; ω = 0.07)	310	59.60%	314	59.90%	241	53.40%	322	57.60%	265	51.60%
											Work status (*p* < .001; ω = 0.15)	*N*	%	*N*	%	*N*	%	*N*	%	*N*	%
Employed	196	37.70%	175	33.50%	113	25.10%	169	30.20%	115	22.40%											
Unemployed	263	50.50%	258	49.20%	263	58.30%	284	50.80%	280	54.50%
Rehabilitation allowance/retired	54	10.40%	74	14.10%	66	14.60%	95	17.00%	112	21.80%
Others	1	0.20%	8	1.50%	3	0.70%	1	0.20%	6	1.20%
No information	6	1.20%	9	1.70%	6	1.30%	10	1.80%	1	0.10%
Diagnoses (ICD-10) (*p* < 0.001; ω = 0.24)	*N*	%	*N*	%	*N*	%	*N*	%	*N*	%
F3 Mood (affective) disorders	321	61.70%	335	63.90%	316	70.10%	356	63.70%	300	58.40%
F4 Neurotic-, stress-related and somatof. disorders	174	33.50%	139	26.50%	85	18.80%	148	26.50%	115	22.40%
F6 Disorders of adult personality and behavior	3	0.60%	26	5.00%	33	7.30%	40	7.20%	67	13.00%
Others	22	4.20%	24	4.60%	17	3.80%	15	2.70%	32	6.20%
At least one psychiatric comorbidity	223	42.90%	259	49.40%	239	53.00%	315	56.40%	353	68.70%
(*p* < .001; ω = 0.17)										
At least two psychiatric comorbidities	71	13.70%	77	14.70%	99	22.00%	107	19.10%	147	28.60%
(*p* < .001; ω = 0.14)										

Note. EMS, early maladaptive schema.

## Discussion

In our study performed in an outpatient rehabilitation setting, the majority of patients showed rather high depression scores [mean BDI-2 score of 26.48 (*SD* = 11.17)] at admission, indicating high symptom burden. Eleven out of the 18 EMSs assessed using the YSQ-S3 explained a high proportion (46%) of variance in depression severity at admission. Thus, both EMSs and domains showed significant predictive power for depression severity as assessed using the BDI-2 at admission, with the strength of the regression coefficients ranging from low to medium. In contrast to our study, Bär et al. (6), focusing on meaningful relationships and patterns for EMSs in various clinical disorders (including depression) beyond personality disorders, identified high EMS scores in Emotional Deprivation (ED), Social Isolation/Alienation (SI), and Emotional Inhibition (E). Similarly, within a systematic review and meta-analysis, Thimm and Chang (7) found the highest EMS scores in depression for Social Isolation/Alienation (SI), whereas the EMS scores were lower for Emotional Deprivation (ED), and, only after exclusion of outliers, did Negativity (NP) show the highest EMS scores. In our study, Social Isolation (SI) scores were, although high, not among the highest schema scores, and Negativity (NP) showed the third highest EMS scores.

This heterogeneity among these studies could at least in part be explained by the heterogeneity of depression, e.g., as a result of comorbidities, different courses of disease, or different environmental factors such as childhood abuse. Nevertheless, the results of our study largely correspond to the literature.

The meta-analysis by Tariq et al. (14) showed a clear association between domains D1 Disconnection, D2 Impaired Autonomy and Achievement, and D4 Other-Directness, while domains D3 Impaired Limits and D5 Exaggerated Vigilance and Inhibition were less strongly associated with symptoms of depression. This meta-analysis included studies on adolescents and young adults, which could at least partly explain the differences in the correlations compared to our study.

In our study, domains D1 Disconnection, D2 Impaired Autonomy and Achievement, and D5 Exaggerated Vigilance and Inhibition showed significant predictive value for predicting BDI-2 scores at admission. The high regression coefficient with D5 can be explained by the clinical sample with a high proportion of depressive disorders. In addition, treatment usually targets specific EMSs rather than domains, and individual EMSs show stronger and more specific associations with psychiatric disorders, therapy outcomes, and coping strategies. We focused on both EMSs and domains since this is in accordance with the schema therapy concept of (3), and, further, both EMSs and domains have been investigated in the literature. Nevertheless, our results support focusing on EMSs rather than on domains in order to investigate psychopathology and treatment outcome.

In a systematic review on the effectiveness of schema therapy for patients with anxiety disorders, obsessive-compulsive disorders, and posttraumatic stress disorders, Peeters et al. (25) provided preliminary evidence that schema therapy may lead to beneficial effects in disorder-specific symptoms and EMSs for those disorders, highlighting significant methodological limitations in current studies. In contrast, a study performed by (26) found that, contrary to predictions, greater initial endorsement of schemas did not predict poor therapy response and that the CBT program was effective for most patients, including patients with high endorsement of maladaptive schemas. A recent study performed by Remmerswaal et al. (27) showed that pre-treatment EMSs and schema modes did not predict symptom improvement, suggesting that Schema Therapy - Cognitive Behavioural Therapy (ST-CBT) is effective regardless of initial schema severity. Similarly, in our study, neither the schemas (EMSs) nor the domains contributed significantly to the prediction of rehabilitation success (measured by symptom reduction in the BDI-2). The variance explanation was marginal at 2%. Among the domains, only D4 Other-Directedness remained in the model for predicting the difference scores in the BDI-2. In accordance with our findings, van Vreeswijk et al. (15) also found this schema domain to be predictive of treatment success.

The rehabilitation patients showed very high levels of schemas SS and US, and the lowest level of schema EM. A cluster analysis including all 18 schemas (EMSs) resulted in differing clusters of rehabilitants with diverging mean schema scores and clinical symptom burden. Interestingly, patients with high schema (EMS) scores showed similar effect sizes with regard to clinical improvement as those with lower schema scores, although patients with high schema scores generally showed higher symptom burden than those with lower schema scores. Thus, although the schema scores were significantly associated with symptom burden, patients with high schema scores at admission showed similar improvement to those with low schema scores at admission. Nevertheless, it has to be pointed out that rehabilitants with high schema scores showed significantly higher symptom burden at admission, and although their clinical improvement was significant, these rehabilitants still showed depression scores of clinical relevance at discharge. Patients with high schema scores assessed rehabilitation success and achievement of therapy goals in a more critical way than patients with low schema scores. Since the majority of the patients were depressed at the time of assessment, this could partly be explained by the negative cognitive triad of depression proposed by A. Beck (28, 29), consisting of a negative view of the self, the world, and the future; however, EMSs were repeatedly shown to be stable traits. Therefore, the fact that they are more critical could also be related to depressive symptoms (and not to EMSs). One can imagine that patients in clusters with stronger EMSs are more critical because they have more severe symptoms at discharge that may hinder them in daily life. A resulting recommendation based on this finding may be to prolong treatment for patients in more severe clusters. Sunde et al. (18) also came to this conclusion in patients with obsessive-compulsive disorder and perceived combined treatment—taking EMSs into account—as a possible therapeutic approach.

We assume that the discrepancies between different studies with regard to the highest EMS scores result in the heterogeneity of the samples resulting from comorbidities, duration of former treatments (that eventually successfully reduced EMSs), and other sample characteristics.

The size of the exploratory, naturalistic sample can be seen as a strength of the study, as it provided an initial impression of the characteristics of EMSs in rehabilitation patients. Limitations of the study arise from the (naturalistic) sample due to the fact that only one rehabilitation facility was included. The study group was relatively homogeneous in that the majority of participants were affected by depression, which was why the Beck Depression Inventory was chosen as the primary outcome. Since it can be assumed that the depressive symptoms overlap with EMSs, such as negativity, the regression analysis could overestimate the influence of EMSs on the symptoms. In further studies, cross-validation could be used to test the model assumptions.

## Conclusion

In our study, EMSs showed a significant influence on the severity of depressive symptoms at admission. However, their impact on treatment success was limited; namely, similar clinical improvement was found in patients with high EMSs. Since patients with high schema scores at admission achieved a significant improvement in depressive symptoms, as did patients with low schema scores at admission, we conclude that all patients can derive clinically relevant benefits from the rehabilitation program, despite a significant interaction effect in the variance analysis. Nevertheless, EMSs may be a significant predictor of psychopathology; that is, high EMS scores can identify patients with a higher risk of future psychopathology. This trend is further supported by the observation that patients in clusters 3, 4, and 5 are more likely to be diagnosed with a personality disorder and are more frequently affected by psychiatric comorbidities. Furthermore, knowledge of EMSs contributes to psychoeducation (e.g., how biography influences current behavior, especially coping modes, facilitating the patient’s understanding of their dysfunctional behavior) and illustrates potentially important targets for further psychotherapeutic treatments.

Since EMS scores are predictors of psychopathology, and high EMS scores can therefore identify patients with a higher risk for future psychopathology, we recommend prolonging the treatment for patients in more severe clusters for long-term stabilization.

## Data Availability

The data analyzed in this study is subject to the following licenses/restrictions: The publication of sensitive health-related data was not covered by informed consent. Requests to access these datasets should be directed to Alexandra.schosser@mail.sfu.ac.at.
