# Supplementary material for: Early maladaptive schemas as predictors of depressive symptoms and treatment success in an outpatient rehabilitation sample
Source: Front Psychiatry. 2026 Jan 28;17:1698633. doi: 10.3389/fpsyt.2026.1698633 (PMC12891196; doi:10.3389/fpsyt.2026.1698633)
Supplement: Supplementary file 1 [file Table1.docx]

Table 1 Appendix Variables excluded from the model for predicting the difference values in the BDI-2 using the EMS

| Model 13 - Excluded Variables  Dependent Variable: difference in BDI-2-scores (discharge – admission) | Beta In | t | Sig. | Partial Corr. | Collinearity Statistics | | |
| --- | --- | --- | --- | --- | --- | --- | --- |
|  |  |  |  |  | Toler-ance | VIF | Minimum Tolerance |
| FA: Failure | -.003 | -.110 | .912 | -.002 | .426 | 2.349 | .388 |
| ED: Emotional Deprivation | -.007 | -.317 | .751 | -.006 | .705 | 1.419 | .474 |
| DS: Defectiveness/Shame | -.015 | -.538 | .591 | -.011 | .478 | 2.094 | .439 |
| MA: Mistrust/Abuse | -.001 | -.050 | .960 | -.001 | .507 | 1.972 | .459 |
| AB: Abandonment/Instability | -.008 | -.307 | .759 | -.006 | .580 | 1.726 | .464 |
| SS: Self Sacrifice | .021 | .945 | .345 | .019 | .798 | 1.254 | .478 |
| EM: Enmeshment/Undeveloped Self | .016 | .668 | .504 | .013 | .629 | 1.590 | .459 |
| EI: Emotional Inhibition | -.027 | -1.073 | .283 | -.021 | .612 | 1.633 | .473 |
| US: Unrelenting Standards/Hypercriticalness | .035 | 1.354 | .176 | .027 | .583 | 1.716 | .476 |
| SI: Social Isolation/Alientation | -.032 | -1.201 | .230 | -.024 | .545 | 1.834 | .471 |
| NP: Negativity | .021 | .742 | .458 | .015 | .468 | 2.139 | .463 |
| VH: Vulnerability to Harm and Illness | -.020 | -.802 | .423 | -.016 | .590 | 1.695 | .452 |

Note. VIF, variance inflation factor. BDI-2 Beck Depression Inventory
